# Acceptability of artificial intelligence for cervical cancer screening in Dschang, Cameroon: a qualitative study on patient perspectives

**DOI:** 10.1186/s12978-024-01828-8

**Published:** 2024-06-28

**Authors:** Malika Sachdeva, Alida Moukam Datchoua, Virginie Flore Yakam, Bruno Kenfack, Magali Jonnalagedda-Cattin, Jean-Philippe Thiran, Patrick Petignat, Nicole Christine Schmidt

**Affiliations:** 1 Faculty of Medicine, University of Geneva, Geneva, Switzerland; 2 Department of Gynaecology and Obstetrics, Dschang Regional Annex Hospital, Dschang, Cameroon; 3 Institute of Global Health, Faculty of Medicine, University of Geneva, Geneva, Switzerland; 4 Signal Processing Laboratory LTS5, School of Engineering, École Polytechnique Fédérale de Lausanne, Lausanne, Switzerland; 5 EssentialTech Centre, École Polytechnique Fédérale de Lausanne, Lausanne, Switzerland; 6 Gynaecology Division, Department of Paediatrics, Gynaecology and Obstetrics, University Hospitals of Geneva, Geneva, Switzerland; 7 Faculty of Social Science, Catholic University of Applied Science, Munich, Germany

**Keywords:** Artificial intelligence, Cervical cancer, Patient acceptability, Patient perspectives, Qualitative study

## Abstract

**Background:**

Cervical cancer is the fourth most frequent cancer among women, with 90% of cervical cancer-related deaths occurring in low- and middle-income countries like Cameroon. Visual inspection with acetic acid is often used in low-resource settings to screen for cervical cancer; however, its accuracy can be limited. To address this issue, the Swiss Federal Institute of Technology Lausanne and the University Hospitals of Geneva are collaborating to develop an automated smartphone-based image classifier that serves as a computer aided diagnosis tool for cancerous lesions. The primary objective of this study is to explore the acceptability and perspectives of women in Dschang regarding the usage of a screening tool for cervical cancer relying on artificial intelligence. A secondary objective is to understand the preferred form and type of information women would like to receive regarding this artificial intelligence-based screening tool.

**Methods:**

A qualitative methodology was employed to gain better insight into the women’s perspectives. Participants, aged between 30 and 49 were invited from both rural and urban regions and semi-structured interviews using a pre-tested interview guide were conducted. The focus groups were divided on the basis of level of education, as well as HPV status. The interviews were audio-recorded, transcribed, and coded using the ATLAS.ti software.

**Results:**

A total of 32 participants took part in the six focus groups, and 38% of participants had a primary level of education. The perspectives identified were classified using an adapted version of the Technology Acceptance Model. Key factors influencing the acceptability of artificial intelligence include privacy concerns, perceived usefulness, and trust in the competence of providers, accuracy of the tool as well as the potential negative impact of smartphones.

**Conclusion:**

The results suggest that an artificial intelligence-based screening tool for cervical cancer is mostly acceptable to the women in Dschang. By ensuring patient confidentiality and by providing clear explanations, acceptance can be fostered in the community and uptake of cervical cancer screening can be improved.

**Trial registration:**

Ethical Cantonal Board of Geneva, Switzerland (CCER, N°2017–0110 and CER-amendment n°4) and Cameroonian National Ethics Committee for Human Health Research (N°2022/12/1518/CE/CNERSH/SP). NCT: 03757299.

## Plain Language Summary

Globally, cervical cancer is the fourth most frequent cancer among women. However, 90% of all deaths caused by cervical cancer occur in low-and middle-income countries. Methods traditionally used in settings like Cameroon to detect cervical cancer unfortunately lack accuracy. Therefore, researchers at the Swiss Federal Institute of Technology Lausanne and the University Hospitals of Geneva are developing an artificial intelligence-based computer aided diagnosis tool to detect pre-cancerous lesions using a smartphone application. The aim of this study was to explore the acceptability and perspectives regarding an AI-based tool for cervical cancer screening for women in Dschang, a city in the west of Cameroon. A qualitative methodology was conducted with six focus groups and a total of 32 participants. The main concerns highlighted by the study are related to privacy, trust in the ability of the healthcare providers, accuracy of the tool as well as the potential negative impact of smartphones. In conclusion, our results show that a computer aided diagnosis tool using artificial intelligence is mostly acceptable to women in Dschang, as long as their confidentiality is preserved, and they are provided with clear explanations beforehand.

## Background

Cervical cancer (CC) represents the fourth most frequent cancer worldwide among women, with 604,000 new cases estimated in 2020. However, the global burden of this disease is unevenly distributed. About 90% of the estimated 342,000 deaths from CC in 2020 occurred in low- and middle-income countries (LMICs) like Cameroon [1]. Without any significant intervention, the World Health Organization (WHO) estimates that deaths linked to CC will increase to 460 000 globally by 2040 with LMICs seeing the greatest relative increase [2].

To combat the increasing burden of CC, WHO adopted a global strategy in November 2020 to accelerate the elimination of CC as a public health problem and set up the following “90–70-90” targets that are to be reached by the year 2030: (i) 90% of girls fully vaccinated by age 15, (ii) 70% of women are screened with a high-performance test by 35 years, and again by 45 years of age, and, (iii) 90% of women identified with cervical disease (precancer and cancer) receive appropriate treatment and management.

More than 95% of CC cases are linked with a persistent human papillomavirus (HPV) infection. While CC is highly preventable, with HPV vaccinations, and screening of precancerous and cancerous lesions, in sub-Saharan Africa (SSA), lack of CC screening and HPV vaccination programmes, alongside a high prevalence of HPV and HIV infections, have contributed to the rising incidence of CC [3].

In high-income countries (HICs), cytology-based screening is a mainstay in CC screening [4]. However, in low-resource settings like Cameroon, WHO recommends primary HPV testing, followed by visual inspection with acetic acid (VIA) and Lugol’s iodine (VILI) for triage of HPV-positive women. VIA is widely used in LMICs due to its low-cost, even if the sensitivity varies between 25% to 94.4% due to the subjectivity of the test depending on the healthcare provider [5].

In 2018, the 3T approach (Test, Triage, Treat), which takes place as a single visit, has been implemented in Dschang, in collaboration with the Cameroon Ministry of Public Health, the Dschang Regional Annex Hospital, and the University Hospitals of Geneva (HUG) [6]. Primary HPV testing consists of HPV self-sampling, carried out by the women themselves (assisted by midwives if necessary) and analysed in about an hour (using the *GeneXpert®*) followed by VIA-triage for HPV-positive women. Finally, if needed, treatment by thermal ablation or LEEP conisation can be performed [7].

Even if recent technical developments have improved early diagnosis of cervical precancerous lesions, accurate visual assessment remains difficult, mainly because of subjectivity and lack of quality control. Currently, the Swiss Federal Institute of Technology Lausanne (EPFL) and HUG are collaborating to develop an automated smartphone-based image classifier that serves as a computer aided diagnosis (CAD) tool for cancerous lesions based on videos obtained only using a smartphone application. The images that are recorded are then classified using an artificial neural network and image processing techniques that distinguish precancerous and cancerous lesions from non-neoplastic cervical tissue [8]. The results are then available on the smartphone application to healthcare professionals and can be shared with patients during consultations to support explanation and thus improve understanding.

While the use of artificial intelligence (AI) in the healthcare field is increasing in recent years, especially in HICs, previous studies have evoked various barriers to the uptake of clinical decision support tools on smartphones by patients and healthcare providers (HCPs). Concerns such as theft of devices, fear of a data breach and perceptions of reduced patient trust were highlighted, though more research is needed to better understand the acceptability of AI as a clinical decision support tool in patients in the Global South [9–11].

The aim of this study is to explore the acceptability and perspectives of females in Dschang, Cameroon, regarding a CAD screening tool for CC relying on AI prior to its implementation in the clinical setting. A secondary objective is to understand in which form and content women would like to receive information about the utilisation of AI for CC screening. Suggestions will be made accordingly to ensure improved acceptance and understanding of AI as a CAD tool for CC screening for future patients and interventions will be proposed considering current literature.

## Methods

### Study site and setting

**Fig. 1:**
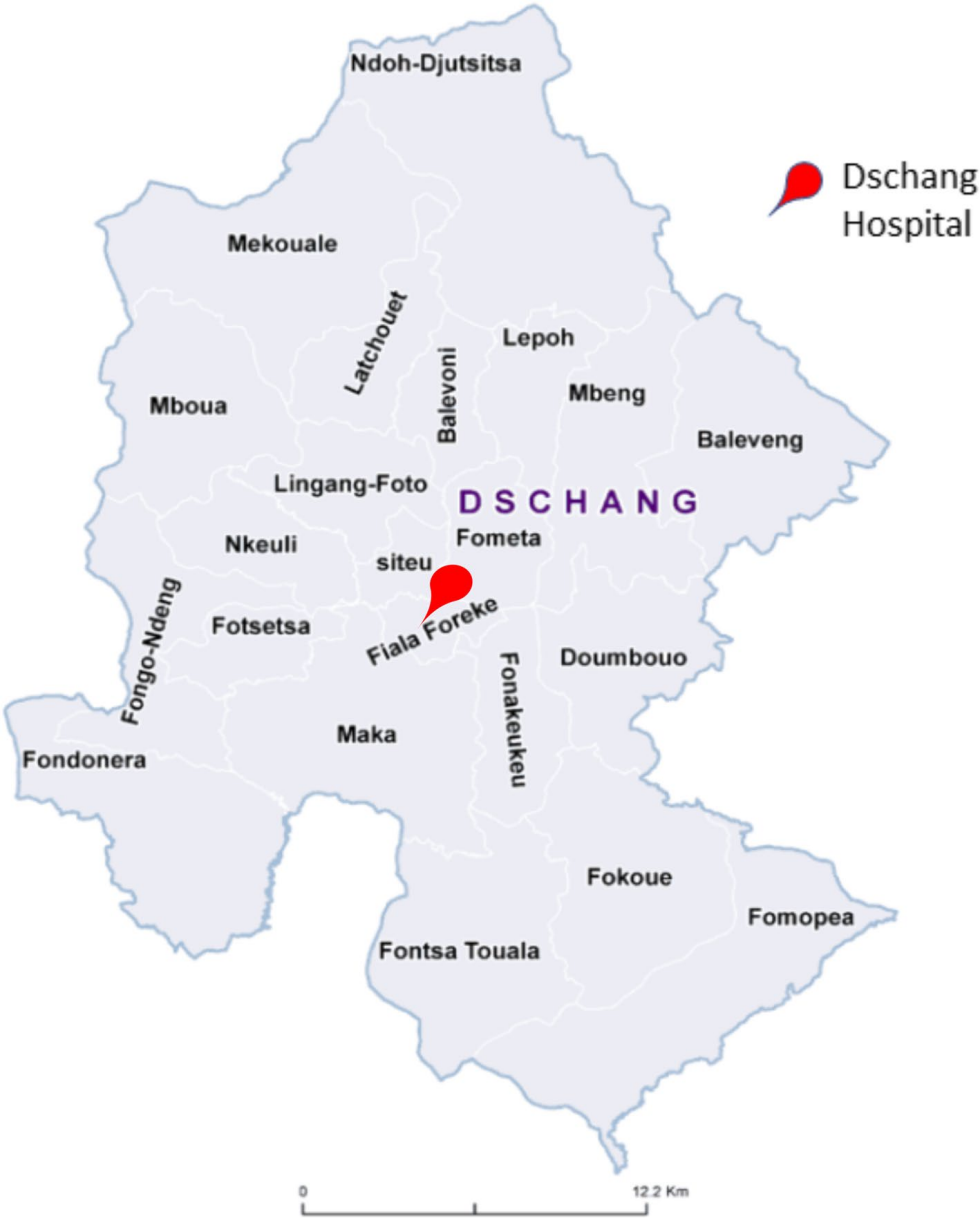
Map of the health areas of the District of Dschang, West Cameroon, modified from Ministère de la Santé Publique du Cameroun (https://dhis-minsante-cm.org/portal/), used from a publication with permission of Datchoua Moukam A.M. [12].

The study took place in the Dschang district, Cameroon in August 2022. The district is composed of Dschang city, an urban area, and the surrounding rural areas, with a total population of approximately 220,000 people (Fig. 1). Females that had already participated in the Dschang Regional Annex Hospital cervical screening programme (3T) were contacted to share their perspective on the use of a screening tool for CC relying on AI.

### Study design

A qualitative methodology using focus groups (FGs) with 4–7 participants was used to gain better insight into the perspectives of women in Dschang on the use of AI for CC screening. While a quantitative methodology allows for standardised results, it does not capture the nuances of participant perspectives that can be collected with a qualitative methodology [13]. Additionally, studies have demonstrated that qualitative methods promote patient engagement in research [14].

A pre-tested semi-structured interview guide based on the current literature on the acceptability of AI and smartphones in patients and HCPs was used to address the following categories:

- Pre-existing understanding of CC.
- Usability and acceptability of smartphone in a medical and personal context.
- Impact of an AI-based diagnosis on patients’ trust in HCPs.
- Usability and acceptability of a smartphone in a medical and personal context.

Women were given an explanation about the use of an AI-assisted clinical decision support tool before being asked to share their perspectives on its use.

### Eligibility criteria and participant recruitment

Women eligible for the 3T-Approach (30–49 years old) who had previously participated in the CC screening program at the Dschang Regional Annex Hospital were contacted by telephone and invited to participate in this study. Mainly females from the rural areas of Mbeng and Fotetsa and the urban area of Fiala-Foreke participated in the study (Fig. 1). The FGs were divided in terms of the participants’ HPV status and level of education, as previous studies have reported a correlation between educational status and health behaviours [15]. Homogeneity with respect to education was chosen to ensure a more free-flowing conversation, where participants did not feel embarrassed to share their perspectives among participants of a higher level of education. Additional socio-demographic characteristics (age, area of residence and marital status) of all participants were also reported. Table 1 summarises the key characteristics of the six focus groups.

**Table 1:** Description of the main characteristics of the participants in all six focus groups

Group number	HPV Status	Level of Education
**1**	Positive; untreated	Primary
**2**		Secondary/Superior
**3**	Positive; treated	Primary
**4**		Secondary/Superior
**5**	Negative	Primary
**6**		Secondary/Superior

### Data collection and analysis

The interviews took place in French and were led by two Cameroonian anthropologists (ADM and VYF) at the Dschang Regional Annex Hospital who have been working on the 3T project since several years. The interviews lasted approximately 60 minutes. Compensation was given to participants to cover their travel costs and snacks. All FGs were audio-recorded after receiving written consent from each participant. They were subsequently transcribed in French, and then analysed using the ATLAS.ti software (version 22), which allows for data storage, management, and qualitative analysis. Qualitative content analysis was used to analyse the transcripts, deductively using a codebook, and complemented by inductive categories. Selected quotations were then translated into English.

### Technology acceptance model

**Fig. 2:**
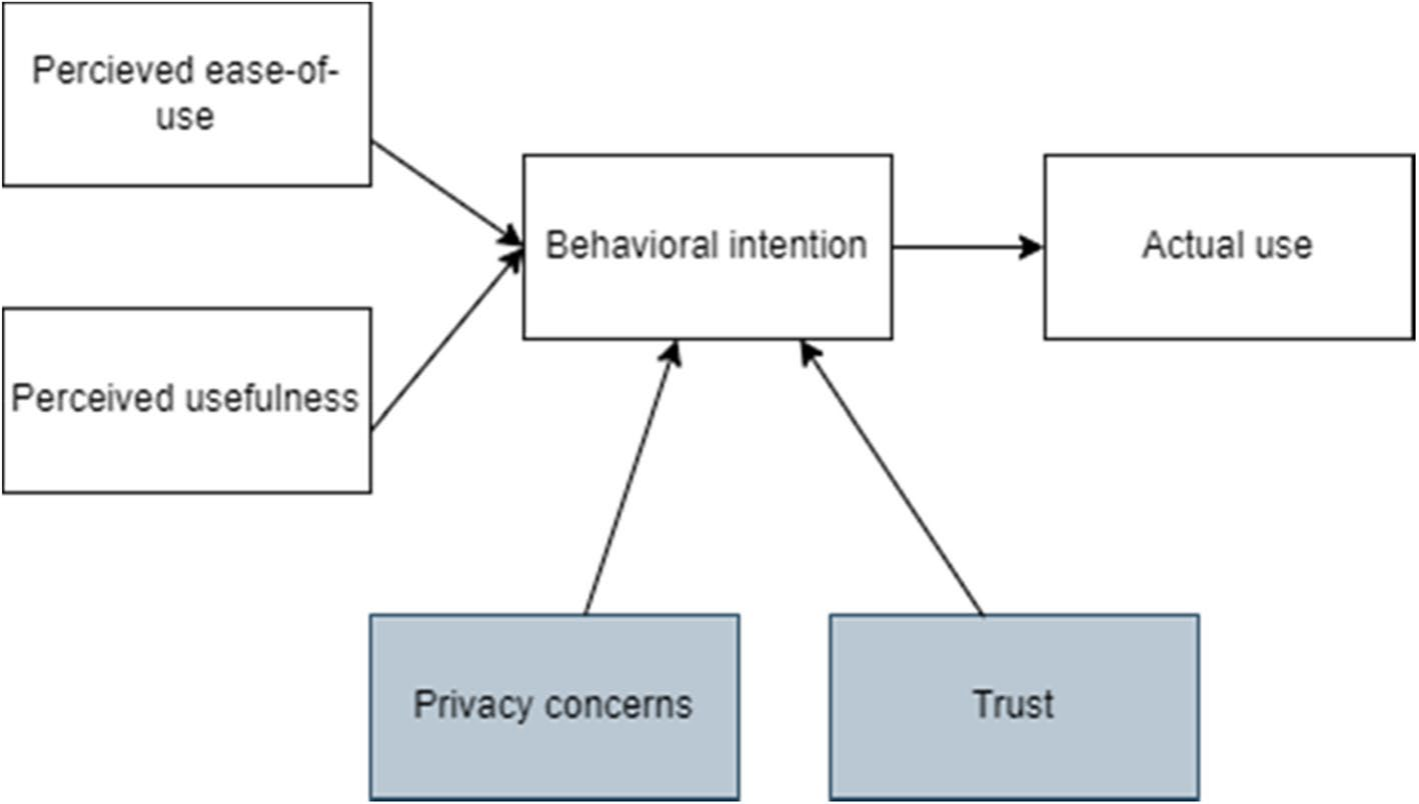
An adapted version of the TAM with the augmentations proposed by Dhagarra et al. in grey

The Technology Acceptance Model (TAM) is a well-known and widely used theoretical framework that aims to explain the various factors influencing an individual’s acceptance and use of technology, notably their perceived usefulness of the technology and perceived ease-of-use. Positive acceptance of these factors is correlated with a positive intention resulting in the actual use of the technology. The model has been studied in various contexts, including healthcare settings, as well as in different target populations, leading to various adaptations and augmentations. In their 2020 study, Dhagarra et al*.* propose an extended version of TAM by additionally integrating trust and privacy concerns as predictors of patients’ acceptance of technology in healthcare delivery settings [16]. That revised framework will be used for further analysis of the patients’ perspectives in the current study. Figure 2 depicts the original model, alongside the modifications proposed by Dhagarra et al. in grey.

## Results

### Socio-demographic characteristics

The six FGs consisted of a total of 32 female participants, aged between 30 and 48 years old. A primary level of education was observed in 38% of the participants, while 40% had a secondary level, and 22% had a tertiary level of education. Most women (94%) claimed to either be in a relationship or married. Finally, 72% of all participants had used a smartphone at least once in their lifetime. Table 2 summarizes the socio-demographic characteristics of the participants.

**Table 2:** Socio-demographic characteristics of participants

**Characteristics**	**Frequency (n)**	**%**
**Level of Education**		
*Primary education*	12	38
*Secondary education*	13	40
*Tertiary education*	7	22
**Marital status**		
*Married*	20	63
*Divorced*	1	3
*In a relationship*	10	31
*Single*	1	3
**Use of smartphones at least once**		
*Yes*	23	72
*No*	9	28
**Age (years)**		
*Range: 30–48*		

### Perceived usefulness and ease-of-use

As described in Fig. 2, perceived usefulness and ease-of-use are two key components of the original TAM. Perceived usefulness can be defined as “the degree to which a person believes that using a particular system would enhance his or her job performance” [17]. From a patient’s perspective, perceived usefulness can also be described as the benefits they expect of the technology. Perceived ease-of-use is defined as “the degree to which a person believes that using a particular system would be free of effort” [16].

Perceived ease of use was not mentioned specifically by the participants. However, participants in all FGs underlined the usefulness of the application, especially in terms of increased efficiency, precision in diagnosis, and facilitation of communication.

*“We say that it [the computer aided diagnosis tool] is good, it facilitates the nurses’ work since the eye cannot visualise well; the device is there to visualise better and it is very fast….” *

(Participant 3, FG2)

Participants also recognised that the use of smartphones facilitated communication between providers, as well as patient-provider communication, since it allowed them to visualise their own cervix and any potential lesions after the gynaecological exam.

Furthermore, two additional factors emerged: trust and privacy concerns. Those were also proposed by Dhagarra et al*.* [16] in their study and our findings from Cameroon are described in the following paragraph.

#### Trust

Three aspects of trust were discussed during the FGs: trust in the accuracy of an AI-assisted diagnosis, trust in the safety of the procedure and trust in the competency of HCPs.

When asked about their level of trust in the accuracy of a diagnostic made using AI, the responses of the participants varied from 50 to 100%. Lack of complete trust in the diagnosis was often attributed to the fact that the system is not necessarily 100% error-free and can malfunction. The competence of HCPs while filming using the smartphone was also evoked as a factor that could influence the accuracy of the diagnosis.

*“I would say 80% in favor because a device can have problems, so we must be sure that the device is in good condition and there is also the user; does the user use it properly? Yes, because for example, if I am a nonprofessional and you give me this, I will take and film as I want! So, you need qualified personnel for that, yes, you need someone qualified.”*

(Participant 2, FG2).

Lack of trust in the safety of smartphones was another important aspect that was highlighted by three out of 32 participants in two different FGs. Participant concerns were related to the dangerous effect smartphones could have on their own health.

*“If I had tested positive, I would not want to be captured by a telephone because I would tell myself that either it aggravates my cancer, or…actually I would not like to be captured by a telephone. Maybe if it were with an ultrasound, but with the phone, knowing that they emit radiations, I would not want to be filmed by a telephone.”*

(Participant 1, FG 5).

Finally, two participants questioned the impact of dependence on the smartphone application on the competency of HCPs. One participant expressed concerns about HCPs being overly reliant on the smartphone and another expressed that HCPs should be away from smartphones while working since they are an important source of distraction.

#### Privacy concerns

Privacy and data protection concerns were a significant point of discussion in each of the six FGs and different perspectives were mentioned. Less than a third of participants, especially smartphone users, feared that their images could potentially be published on social media pages, like Facebook.

*“A risk is the protection of data of the patients mainly. Yes! You keep them in the phone, you..you guarantee protection, but it is a phone. Artificial intelligence or not, once it is connected, data is no longer protected from what I know.”*

(Participant 2, Focus Group 4)

Most participants were less concerned about the protection of their data and believed that even if their photos were published, their identities would remain protected. The absence of these concerns was closely related to confidence in HCPs, highlighting that upholding patient confidentiality was the HCP’s responsibility.

*“I too have trust since in this profession, confidentiality is a must and I think that you truly have to be someone with a certain immorality to publish such things. I have confidence. Since the beginning when they spoke to me of this, I accepted.”*

(Participant 2, FG2)

#### Informational needs

The secondary objective of this study was to understand the type and form of information women would like to receive about the utilisation of AI for CC screening. About a third of women were satisfied to know their result of the HPV-test and did not require further information about the use of AI, as explained by a woman from a secondary focus group:

*“For me, nothing [no information] in particular. I am just waiting for the results, that they first have a look at the photos with the old method [without AI], that they perhaps know if there really are or not the lesions, and that we then use this artificial [intelligence] method, and if all is still good, I am ok. I am just waiting for the results, nothing else.”*

(Participant 1, FG4).

On the other hand, a few women wanted to better understand how the smartphone application worked and whether they themselves could use it.

*“I would just like to know the specifics, actually understand the artificial intelligence, what it is going to do....what is artificial intelligence. For example, when we talk about an application, we give its advantages, its limits, how it leads to the result that we expect […] Just give some understandable information about the app itself. So that I know what the artificial intelligence that analyzed my data is called, how it reacts. It is always a matter of trust and knowledge too.”*

(Participant 2, FG4).

With respect to the form of information, participants in all six FGs stated that they would prefer a face-to-face conversation with HCPs.

*“Yes, a conversation face-to-face because when you discuss with her [the HCP], you can ask questions and she can respond.”*

(Participant 2, FG1).

In addition, a few women mentioned that they would benefit from an additional explicative video or a written explanation, as a brochure.

*“I prefer a brochure and an explanation face-to-face. A brochure so that I can keep the information for myself and when I am not at the hospital, I can still inform myself and research what is written. And the explanation because there are little subtilities that the person that explains knows best and it perhaps also allows us to ask questions on what is written so for me the two methods should be used.”*

(Participant 2, FG4).

#### Influence of educational status on the factors influencing the acceptance of AI-based diagnostics

As the FGs were organized according to the participant’s educational level, the influence of education on the previously described factors was explored. Most women of primary and superior levels of education saw the tool as an advanced use of technology and were curious about its use.

*“We are very grateful for the fact that science is making progress. When it does, it means that we can quickly find our cure!”*

(Participant 1, FG1).

Furthermore, they trusted the CAD tool, mainly because of its increasing precision. However, even if a minority of women mainly with a primary level of education stated it was a “Western influence” that they were unable to understand, women in the FGs with a primary education level mainly expressed their confidence in the tool.

*“We have complete trust if they don’t publish [our photos] in any which way.”*

(Participant 2, FG1).

Overall, while the barriers mentioned by both groups were similar, including confidentiality concerns and trust, women with a higher educational status exhibited a greater tendency to question the reliability of the tool. Hereby, women with a higher level of education, who were also more experienced smartphone users, discussed concerns regarding potential smartphone malfunctions, the impreciseness of the tool and the inexperience of HCPs.

## Discussion

**Fig. 3:**
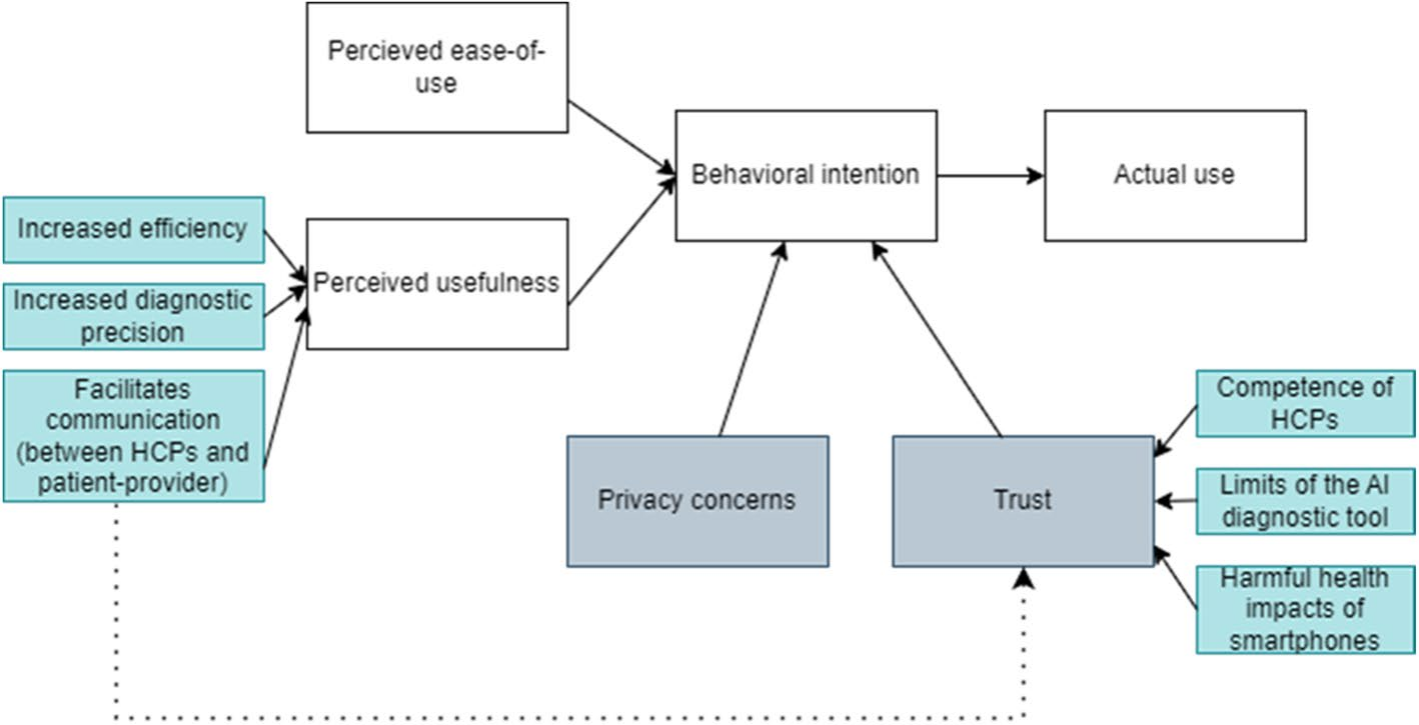
Summary of the factors explored during the study (denoted in blue) using the modified TAM

The following discussion section will primarily address key findings that are important for HCPs to consider, when using a smartphone including AI-aided CAD tools, to avoid negative impacts on the uptake of CC screening by the women or on return to follow-up. In this study, the acceptance of women in Dschang regarding a CAD tool for CC relying on AI was studied. The TAM was identified as an appropriate model to investigate the various factors influencing the acceptance of the AI-based diagnostic aid. We identified that, in the selected study setting, patients’ acceptance was mainly impacted by perceived usefulness, privacy concerns and trust. Perceived ease-of-use was not a main concern probably because patients are not end-users of the application themselves and so are unaffected by its usability. The various themes that were revealed during the study were categorized according to the TAM, as depicted in blue in Fig. 3.

Overall participants perceived the AI-based CAD tool on a smartphone as a support for enhancing the diagnoses of CC. The findings can be compared in theory on two distinct levels: first, the use of the smartphones to diagnose CC and second, the use of AI to diagnose the disease. However, many participants in the study made no difference between the use of smartphones and an AI-based technology. Nevertheless, the following section will highlight important factors and differences between their perceptions related to the use of smartphones and AI-based technology when possible.

### Acceptability of smartphones

Regarding the acceptability of smartphones, a study by Mungo et al*.*, showed that smartphone-based cervicography is highly acceptable in HPV-positive women living with HIV in Western Kenya [17]. This observation was confirmed in our study, but it is important to point out that a few women expressed apprehension about the radiation emitted by smartphones and thus were less inclined to accept the method. Similar findings were encountered in another study in Kenya that analysed HCPs and patients’ perspectives on using an mHealth tool for eye care [10]. Indeed, in this study, a minority of patients were concerned about the negative health impact of mobile phones and therefore preferred traditional methods instead. Even if this perception was not shared by most women in our settings, it is important that HCPs are aware of this barrier. In a study aiming to understand and prevent health concerns about emerging mobile health technologies, Materia et al*.* highlighted the importance of evaluating and addressing health concerns related to use of smartphones and developing evidence-based communication strategies to limit them [18].

Professionals, therefore, need to be trained adequately to address safety concerns, including misconceptions, prior to the implementation of new technologies. Effective patient-centred communication can help recognise and address these concerns. Moreover, misconceptions especially need to be explored in future studies to better understand their potential impact on CC screening.

### Acceptability of AI-based CC screening

Since AI-based CC screening is a novel method, there are limited studies concerning the acceptability of this technique by patients, especially in LMICs. In this study, a few women, especially those with a higher level of education, exhibited reservations about the AI-based tool’s potential to malfunction (either due to issues in the smartphone or the impreciseness of the AI). Some also expressed concerns of the inexperience of the HCP handling the tool. However, most women viewed the AI-based tool as an enhanced method to improve diagnostic precision of HCPs.

Firstly, it needs to be acknowledged that our results are in line with the current literature on the use of AI in the field of cancer diagnosis. Studies report that patients tend to accept AI more easily if a dangerous disease such as cancer can be avoided [19]. Other studies exploring the perceptions of AI in the diagnosis of breast and skin cancer concluded that it was an acceptable adjunctive technology for patients, if the provider-patient relationship was preserved, but not an acceptable substitute for physicians or radiologists [15, 20, 21]. It is important to note, however, that these studies were conducted in high-income countries, i.e. the United Kingdom, United States, and Italy and no studies in SSA could be identified.

With respect to HCP competence concerns encountered in our study, similar perspectives were reported by other studies exploring the acceptability of similar CAD tools in various healthcare domains. A study in Uganda regarding HCPs’ perspectives on the acceptability of a mobile health tool revealed a concern that using a mobile application in front of patients and their families would undermine their trust in the HCP’s ability to diagnose and treat [9]. This perception was echoed in another study in the UK where HCPs were worried that using mobile tools during patient interactions would be perceived as unprofessional [11]. However, our findings demonstrate that if the usage of smartphones is explained to the patients beforehand, most participants had continued trust and confidence in the HCPs as well as the diagnosis made by the AI-based tool. Only a minority of participants thought that the usage of this smartphone application would make HCPs overly reliant, distracted, or lazier. Nonetheless, HCPs need to be adequately trained on how to communicate the inclusion of the AI-device during patient consultations, to avoid misperceptions regarding its use.

Importantly, regarding communication, most participants recognized that smartphones are an important tool to facilitate communication between HCPs and patients. By showing the images of their cervix to patients, HCPs can promote transparency and thus establish trust and reinforce patient-provider communication.

### Patient confidentiality

The last factor that is important to address is privacy and confidentiality concerns, which were revealed across all groups regardless of their education level. Concerns regarding videos being posted on social media were highlighted, especially when smartphones are used by students or trainees. These concerns have been underlined in previous studies, such as a meta-synthesis of qualitative studies evaluating public perceptions of AI in healthcare, mostly conducted in the Global North [22]. However, these concerns were highlighted to a lesser extent in studies based in the Global South. A study based in Bangladesh reported that privacy concerns had an insignificant impact on the adoption of eHealth [23]. Similarly, a study by Asgary et al. on the CC screening with smartphones in Ghana reported minimal privacy concerns [24]. Finally, in a systematic review analysing barriers to the use of mobile health in developing countries, privacy and confidentiality concerns were evoked only 3% of the time [25]. However, HCPs should address these concerns by explaining to the patients procedures of data storage and handling of data protection issues.

In summary, to address factors affecting the acceptance of either smartphones or AI-based technology, patients need to be informed adequately by HCPs. Hereby, participants unanimously agreed that an explanation face-to-face with the HCPs would be the best way to provide information regarding the AI-based tool, as it would allow them to ask questions and receive the information that they need. Additionally, it should be acknowledged that some participants (especially those with higher education) were interested in additional means of information, such as brochures or videos.

### Strengths and limitations

Although this study is, to the best of our knowledge, one of the first studies in Cameroon that analyses the acceptability of AI for CC screening and one of the few in SSA, some limitations need to be acknowledged. First, an interviewer or selection bias cannot be excluded. As some FGs were conducted in larger groups of 7 participants, participants’ ability to express their opinions freely might be affected. To mitigate this, FGs were limited to women of similar educational backgrounds and were conducted by a Cameroonian anthropologist. However, the anthropologist’s higher level of education may have influenced participants’ responses, especially in FGs of participants with a primary level of education.

Moreover, even if the FGs were comprised of women of diverse socio-economic backgrounds, the participants had already taken part in the CC screening program and therefore may have systematically included those who are inclined towards prevention and have already been sensitized to the importance of CC screening. Furthermore, the study revealed that the acceptability of an AI-based tool can be influenced by the utilisation of smartphones, highlighting the need to explore differences affecting acceptability in larger qualitative and quantitative studies. Additionally, the use of the TAM can be seen as simplistic, and its suitability can be debated, since it is primarily intended for end-users of the technology, which the patients were not in our study. However, in our opinion, using the TAM allowed us to classify and understand existing barriers and facilitators to AI-based diagnostics.

The last limitation can be seen in the methodology of the study. A qualitative methodology was chosen as an appropriate approach since it allowed us to explore the perspectives of females and capture insights into the ways people perceive and interpret their surroundings [14, 26]. But qualitative studies have limited generalizability. However, as saturation was achieved for most themes such as privacy concerns and perceived usefulness, we consider the results of the study to be important for similar settings.

Given the strengths and limitations of our study, further research is needed. Hereby, the following three areas of research seem important. Firstly, to gain a broader insight into patients’ acceptability of AI, a quantitative analysis should be considered exploring the influence of women’s educational status, but also including the perspectives of spouses and other community members, since they have a significant impact on patients’ attitudes. Secondly, misconceptions about the dangers of smartphones in the community should be explored. Finally, acceptability should also be assessed after an initial pilot phase of the AI tool in CC screening, since perspectives may shift after the experience.

## Conclusions

Overall, our findings suggest that an AI-assisted screening tool for CC can therefore be seen as largely acceptable to women in Dschang, irrespective of their socio-economic status and level of education. However, acceptability is significantly contingent on preserving the confidentiality and privacy of the images or videos taken.

We therefore recommend that all participants receive a counselling session before the AI-based screening that informs the patients about the steps of the procedure, the purpose, and advantages of the tool, as well as potential risks that are associated with it. Explanations related to how the photos and videos will be stored should be provided as well as an assurance that the images taken will solely be for the use of other HCPs. It would also provide the patients with an opportunity to ask questions if needed.

HCPs, therefore, need to be trained in effective patient-centred communication. By ensuring patient confidentiality and by providing clear explanations, acceptance of this method can be fostered in the community, thereby improving the uptake of CC screening, and reducing the burden of CC in LMICs.

In summary, based on the results of our study, the following three suggestions can be made to HCPs when they introduce this CAD tool for CC screening to patients:

1. Emphasize that the AI-assisted tool is used to provide information about and to assist the diagnosis.
2. Ensure the protection of patients’ data and provide the patients with assurance regarding its strict confidentiality.
3. Convey the purpose, benefits, and potential risks of the tool in written form (brochures) for patients who would like to learn more about the tool.

This AI-assisted diagnosis tool could improve CC screening in Dschang and therefore reduce the burden of this women's health concern in the region. However, the successful implementation of this tool hinges on acceptance from both HCPs and patients. This study indicates that patients tend to be open to AI-based solutions.

## Data Availability

Datasets (transcripts) are not publicly available due to the sensitivity of the data, but the interview guide or summaries of transcripts (including categories and codes) can be made available from the corresponding author upon reasonable request.

## Abbreviations

CC: Cervical cancer
LMIC: Low-and-middle income country
WHO: World Health Organization
HPV: Human papillomavirus
SSA: Sub-Saharan Africa
HIC: High-income country
VIA/VILI: Visual inspection with acetic acid/ Lugol’s iodine
HUG: University hospitals of Geneva
EPFL: Swiss federal institute of technology lausanne
CAD: Computer-aided diagnosis
AI: Artificial intelligence
HCP: Healthcare provider
FG: Focus group
TAM: Technology acceptance model

## References

[1] Cervical cancer. https://www.who.int/news-room/fact-sheets/detail/cervical-cancer. Accessed 29 July 2022.

[2] Cervical cancer. In: WHO | Regional Office for Africa. https://www.afro.who.int/health-topics/cervical-cancer. Accessed 1 Aug 2022.

[3] Jedy-Agba E, Joko WY, Liu B (2020). Trends in cervical cancer incidence in sub-Saharan Africa. Br J Cancer.

[4] Indicator Metadata Registry Details. https://www.who.int/data/gho/indicator-metadata-registry/imr-details/3240 .Accessed 9 Aug 2023.

[5] Viñals R, Jonnalagedda M, Petignat P, Thiran JP, Vassilakos P (2023). Artificial Intelligence-Based Cervical Cancer Screening on Images Taken during Visual Inspection with Acetic Acid: ASystematic Review. Diagnostics (Basel)..

[6] Vassilakos P, Tebeu P-M, Halle-Ekane GE, Sando Z, Kenfack B, Baumann F, Petignat P (2019). Vingt années de lutte contre le cancer du col utérin en Afrique subsaharienne - Collaboration médicale entre Genève et Yaoundé. Rev Med Suisse.

[7] Grohar D, Vassilakos P, Benkortbi K, Tincho E, Kenfack B, Petignat P (2020). Scaling up community-based cervical cancer screening in Cameroon employing a single visit approach. International Journal of Gynecologic Cancer.

[8] Baleydier I, Vassilakos P, Viñals R (2021). Study protocol for a two-site clinical trial to validate a smartphone-based artificial intelligence classifier identifying cervical precancer and cancer in HPV-positive women in Cameroon. PLoS ONE.

[9] Ellington LE, Najjingo I, Rosenfeld M (2021). Health workers’ perspectives of a mobile health tool to improve diagnosis and management of paediatric acute respiratory illnesses in Uganda: a qualitative study. BMJ Open.

[10] Lodhia V, Karanja S, Lees S, Bastawrous A (2016). Acceptability, Usability, and Views on Deployment of Peek, a Mobile Phone mHealth Intervention for Eye Care in Kenya: Qualitative Study. JMIR Mhealth Uhealth.

[11] Patel R, Green W, Shahzad MW, Larkin C (2015). Use of Mobile Clinical Decision Support Software by Junior Doctors at a UK Teaching Hospital: Identification and Evaluation of Barriers to Engagement. JMIR Mhealth Uhealth.

[12] Datchoua Moukam AM, Embolo Owono MS, Kenfack B, Vassilakos P, Petignat P, Sormani J, Schmidt NC. "Cervical cancer screening: awareness is not enough". Understanding barriers to screening among women in West Cameroon-a qualitative study using focus groups. Reprod Health. 2021;18(1):147. 10.1186/s12978-021-01186-9. 10.1186/s12978-021-01186-9 PMC8268254 34243778

[13] Gibbons C, Singh S, Gibbons B, Clark C, Torres J, Cheng MY, Wang EA, Armstrong AW (2018). Using qualitative methods to understand factors contributing to patient satisfaction among dermatology patients: a systematic review. J Dermatol Treat.

[14] Rolfe DE, Ramsden VR, Banner D, Graham ID (2018). Using qualitative Health Research methods to improve patient and public involvement and engagement in research. Res Involv Engagem.

[15] Pesapane F, Rotili A, Valconi E (2023). Women’s perceptions and attitudes to the use of AI in breast cancer screening: a survey in a cancer referral centre. BJR.

[16] Dhagarra D, Goswami M, Kumar G (2020). Impact of Trust and Privacy Concerns on Technology Acceptance in Healthcare: An Indian Perspective. Int J Med Informatics.

[17] Mungo C, Osongo CO, Ambaka J, Randa MA, Samba B, Ochieng CA, Barker E, Guliam A, Omoto J, Cohen CR (2021). Feasibility and Acceptability of Smartphone-Based Cervical Cancer Screening Among HIV-Positive Women in Western Kenya. JCO Glob Oncol..

[18] Materia FT, Faasse K, Smyth JM (2020). Understanding and Preventing Health Concerns About Emerging Mobile Health Technologies. JMIR Mhealth Uhealth.

[19] McCradden MD, Sarker T, Paprica PA (2020). Conditionally positive: a qualitative study of public perceptions about using health data for artificial intelligence research. BMJ Open.

[20] Lim K, Neal-Smith G, Mitchell C, Xerri J, Chuanromanee P (2022). Perceptions of the use of artificial intelligence in the diagnosis of skin cancer: an outpatient survey. Clin Experimental Derm.

[21] Nelson CA, Pérez-Chada LM, Creadore A (2020). Patient Perspectives on the Use of Artificial Intelligence for Skin Cancer Screening. JAMA Dermatol.

[22] Wu C, Xu H, Bai D, Chen X, Gao J, Jiang X (2023). Public perceptions on the application of artificial intelligence in healthcare: a qualitative meta-synthesis. BMJ Open.

[23] Hoque MR, Bao Y, Sorwar G (2017). Investigating factors influencing the adoption of e-Health in developing countries: A patient’s perspective. Inform Health Soc Care.

[24] Asgary R, Cole H, Adongo P, Nwameme A, Maya E, Adu-Amankwah A, Barnett H, Adanu R (2019). Acceptability and implementation challenges of smartphone-based training of community health nurses for visual inspection with acetic acid in Ghana: mHealth and cervical cancer screening. BMJ Open.

[25] Kruse C, Betancourt J, Ortiz S, Valdes Luna SM, Bamrah IK, Segovia N (2019). Barriers to the Use of Mobile Health in Improving Health Outcomes in Developing Countries: Systematic Review. J Med Internet Res.

[26] Morgan DL (2012). The SAGE Handbook of Interview Research: The Complexity of the Craft.

